# Protocol for multispectral imaging on cryosections to map myeloid cell heterogeneity in its spatial context

**DOI:** 10.1016/j.xpro.2023.102601

**Published:** 2023-09-23

**Authors:** Elias B. Wieland, Laura J.A.P. Kempen, Chang Lu, Marjo M.P.C. Donners, Erik A.L. Biessen, Pieter Goossens

**Affiliations:** 1 Cardiovascular Research Institute Maastricht, Experimental Vascular Pathology, Department of Pathology, Maastricht University Medical Center+, Maastricht, The Netherlands; 2 Laboratory of Immunology and Vaccinology, Faculty of Veterinary Medicine, FARAH, Liège University, Liège, Belgium; 3 Laboratory of Immunophysiology, GIGA Institute, Liège University, Liège, Belgium; 4 Institute for Computational Biomedicine, Heidelberg, Germany; 5 Institute for Molecular Cardiovascular Research, RWTH Aachen University, Aachen, Germany

**Keywords:** Single Cell, Immunology, Microscopy, Molecular Biology, Antibody

## Abstract

Recent technical advances, such as single-cell RNA sequencing and mass cytometry, improve identification of cell types and subsets in a range of healthy and diseased tissues at the expense of their cellular and molecular context. Here, we present a protocol for *in situ* multispectral imaging to map myeloid cell heterogeneity in tissue cryosections, describing steps for cutting sequential sections, antibody titration, and building a spectral library. We then detail procedures for multispectral imaging and preparing data for downstream analysis.

For complete details on the use and execution of this protocol, please refer to Goossens et al. (2022).^1^

## Highlights

• Single-shot approach for immunofluorescent multiplex imaging
• A pipeline for spectral library composition before multispectral imaging
• Various approaches to reduce tissue autofluorescence including bleaching
• An open-access script to allow alignment of histological and immunofluorescent images

## Abstract (editor highlights)

Publisher’s note: Undertaking any experimental protocol requires adherence to local institutional guidelines for laboratory safety and ethics.

## Graphical abstract

**Figure:**
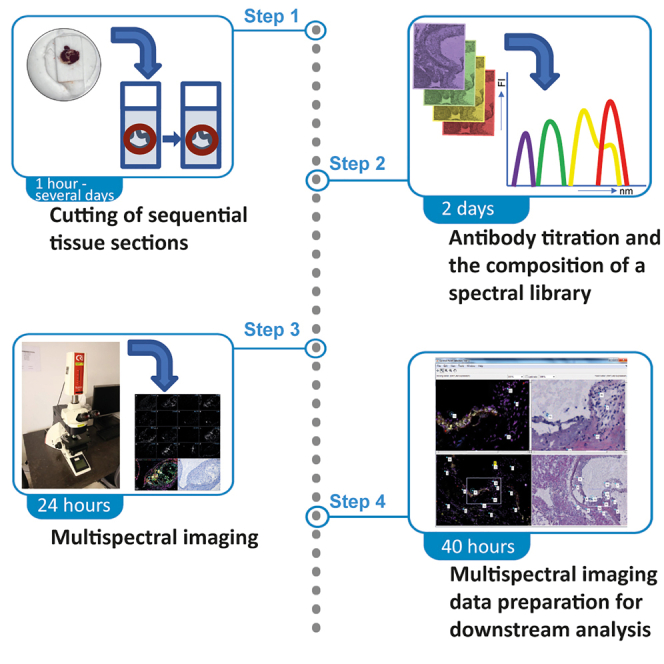

## Before you begin

This protocol describes a comprehensive workflow for multiplex fluorescent imaging based on a spectral library, followed by instructions to prepare the data for downstream image analysis. While we here illustrate its utility on myeloid cell heterogeneity mapping in murine atherosclerotic plaques, the protocol has already been validated on a broad variety of other tissues, both human and murine, including different tumors, spleen, lymph node, lung and liver.

After collection of the tissue and embedding in Optimal Cutting Temperature medium (OCT), tissue sections are cut with a cryostat (Step: cutting of sequential tissue sections).

Before acquiring the multi-marker image, a panel of antibodies coupled to dyes that have similar excitation, but differential emission spectra must be selected. These spectra are assembled into a dye-coupled antibody panel, the so-called spectral library (Step: antibody titration and the composition of a spectral library).

Next, multi-marker images are acquired from antibody panel-stained sections (Step: Multiplex imaging). These can be deconvoluted into the individual spectra based on the spectral library. Thus, multispectral imaging is a single-shot approach bypassing the time-consuming and laborious sequential staining methods that often compromise antigen and fluorochrome stability as well as tissue integrity over the course of several staining cycles. Finally, H&E staining is performed on the fluorescently imaged tissue section to allow histopathological evaluation of the tissue and to facilitate cell segmentation. The spatial coordinates of thus identified cells can then be projected onto the corresponding fluorescent images and the intensities of the individual markers extracted (Step: multispectral imaging data preparation for downstream analysis). The acquired images can be further analyzed using our in-house developed image analysis pipeline.^1^

***Note:*** Combination of this multispectral imaging approach with mass spectrometry imaging (MSI) or spatial transcriptomics on the same or an adjacent tissue section allows to further define the cells’ phenotype and subsets’ microenvironment. These flanking tissue sections must be collected on slides suitable for these applications. We did not elaborate on the detailed methods for these parallel approaches here but we have previously described an example on how to integrate the multimodal data layers.^1^

### Institutional permissions

The examples illustrating the protocol were collected from animal experiments approved by a local Committee for Animal Welfare (IvD Maastricht University, #2014-071) and performed under the Maastricht University animal facility’s standard conditions, in accordance with Dutch national laws and regulations.

### Preparation of required buffer

**Timing: 15 min**

1. Preparation of blocking buffer (PBS+ 4% FCS).
  a. 2 mL of Fetal Calf Serum (FCS) + 48 mL 1× PBS.
  b. Filter through a 0.2 μm filter.

***Note:*** Store at 4°C for up to two weeks.

## Key resources table

The following table contains all relevant resources used to perform multispectral imaging and preparation of image data for subsequent analysis.

**Table:** 

REAGENT or RESOURCE	SOURCE	IDENTIFIER
**Antibodies**		

Anti-CD11b – Brilliant Blue 700 (clone M1/70), 1:40	BD Biosciences	RRID: AB_2744272
Anti-CD44 – PE/Cy5 (clone IM7), 1:500	BD Biosciences	RRID: AB_10896296
Anti-CD47 – Brilliant Violet 480 (clone miap301), 1:20	BD Biosciences	RRID: AB_2743842
Anti-CD68 – Brilliant Violet 605 (clone FA-11), 1:100	BioLegend	RRID: AB_2616811
Anti-CD107b – PerCP/Cy5.5 (clone M3/84), 1:30	BD Biosciences	RRID: AB_2738980
Anti-CD206 – Brilliant Violet 650 (clone C068C2), 1:30	BioLegend	RRID: AB_2562445
Anti-Dectin1 – PerCP/eFluor 710 (clone bg1fpj), 1:30	Thermo Fisher Scientific	RRID: AB_2573779
Anti-F4/80 – eFluor 506 (clone BM8), 1:40	Thermo Fisher Scientific	RRID: AB_2637190
Anti-Ki67 – eFluor 450 (clone SolA15), 1:20	Thermo Fisher Scientific	RRID: AB_11151155
Anti-Ly6G – Brilliant Violet 570 (clone 1A8), 1:20	BioLegend	RRID: AB_10899738
Anti-MHC-II – PE/eFluor 610 (clone M5/114.15.2), 1:400	Thermo Fisher Scientific	RRID: AB_2574618
Anti-Perilipin2 – Alexa Fluor 488 (polyclonal), 1:20	Novus Biologicals	RRID: AB_787904
Anti-MerTK – Super Bright 702 (DS5MMER), 1:20	eBioscience	RRID: AB_2717174
Anti-CD205 – Super Bright 436 (205yekta), 1:40	Thermo Fisher Scientific	RRID: AB_2637130

**Biological samples**		

Female *ldlr*^−/−^ mice fed a Western-type diet for 10 weeks before sacrifice	Jackson Laboratory	https://www.jax.org/strain/002207

**Chemicals, peptides, and recombinant proteins**		

Western-type diet (“diet W”)	SDS Diets	N/A
PBS	Cytiva	Cat# SH30028.02
Fetal calf serum	Serana	Cat# S-FBS-SA-015
Syringe filter 0.22 μm	MilliporeSigma	Cat# SLGPR33RB
Acetone	Merck	Cat# 1000145000
Isopentane	VWR	Cat# 24872.323
7-AAD	BD Biosciences	RRID: AB_2869266
OCT compound	VWR	Cat# 361603E
Cryomold	Polysciences	Cat# 18985-1
ProLong Gold Antifade Mountant	Thermo Fisher Scientific	Cat# P36930
Hematoxylin	Klinipath	Cat# VWRK4085.9001
Eosin	VWR/Q Path Chemicals	Cat# 10047101
TrueBlack lipofuscin autofluorescence quencher	Biotium	Cat# 23007
Sudan Black B	Sigma-Aldrich	Cat# 199664

**Software and algorithms**		

Nuance 3.0.2	PerkinElmer	https://www-punchout.perkinelmer.com/Content/LST_Software_Downloads/tissueimaging/NuanceUserManual_3_0_2_rev0.pdf
Fluorescence SpectraViewer	Thermo Fisher Scientific	https://www.thermofisher.com/order/fluorescence-spectraviewer#!/
MATLAB	MathWorks	https://www.mathworks.com/products/matlab.htm
QuPath	QuPath	https://qupath.github.io/
StarDist QuPath extension	Schmidt et al.^2^	https://qupath.readthedocs.io/en/0.4/docs/deep/stardist.html
ImageJ	ImageJ	https://imagej.nih.gov/ij/
Nuance image and H&E co-registration code	MATLAB	https://github.com/ChangLu92/CoRegi-Muitispectral-HE

**Other**		

Leica DM4000 B LED	Leica	N/A
HC PLAN APO 20×/0.70 PH 2 (air) objective	Leica	N/A
Nuance FX camera	PerkinElmer	N/A
Filter cubes I3, N2.1, A, S blue/aqua	Leica	N/A
Leica EL6000 external light source	Leica	N/A
Klinipath Silan microscopy slides	VWR	KLINKP-SIL-3049
ITO-coated slides	Delta Technologies	Cat# CG-40IN-S115
Mini PAP pen	Thermo Fisher Scientific	Cat# 008877
Custom-designed bleaching device	N/A	N/A
LED bleaching panels	N/A	N/A
Cryostat, CM 3050 S	Leica	N/A

## Step-by-step method details

### Cutting of sequential tissue sections

**Timing: 1 h to several days**

The first step describes sample preparation and storage of the tissue sections. The estimated time for cutting depends on the cohort size and experience.

1. Tissue collection and sectioning.
  a. Collect fresh, unfixed tissue samples.
  b. Place the tissue, with the cutting plane down, in a labelled cryomold and add OCT compound until the tissue is completely covered.
  c. Transfer the cryomold into a dry ice-cooled container of isopentane and let it float until the OCT has solidified.
  d. Store the cryomold on dry ice until all samples are collected and store them at −80°C in sealed bags or wrapped in aluminum foil to minimize drying out.
  e. Cut 7 μm thick cryosections (e.g., with Cryostat, CM 3050, Leica) and collect them on sequential microscopy slides.
    ***Note:*** Sequential cutting will furnish flanking tissue sections, positioned on consecutive slides, to enable complementary analyses to the multispectral image, such as immunohistochemistry, mass spectrometry or spatial transcriptomics.
  f. Dry the slides in a desiccator for several hours at room temperature (at around 20°C) before storing them at −80°C (for up to several months).
    **CRITICAL:** It is important to air-dry the tissue sections completely before freezing to prevent tissue damage through crystallization.
    **CRITICAL:** For mass spectrometry imaging, conductive indium tin oxide (ITO) slides are used to mount the tissue sections. The use of OCT medium for embedding is not advisable as residues of OCT may remain on the tissue sections and cause analyte signal suppression in mass spectrometry imaging through competition with tissue compounds for ions during the ionization process.^3^ Instead, tissue sections can be cut from non-embedded snap frozen tissue. Cut tissue sections on ITO slides should be kept at −20°C and not be dehydrated. For spatial transcriptomics, continue with specific slides and/or collect tissues in a confined area of the slide, as recommended by the respective manufacturer.

### Antibody titration and the composition of a spectral library

**Timing: 2 days**

**Figure 1:**
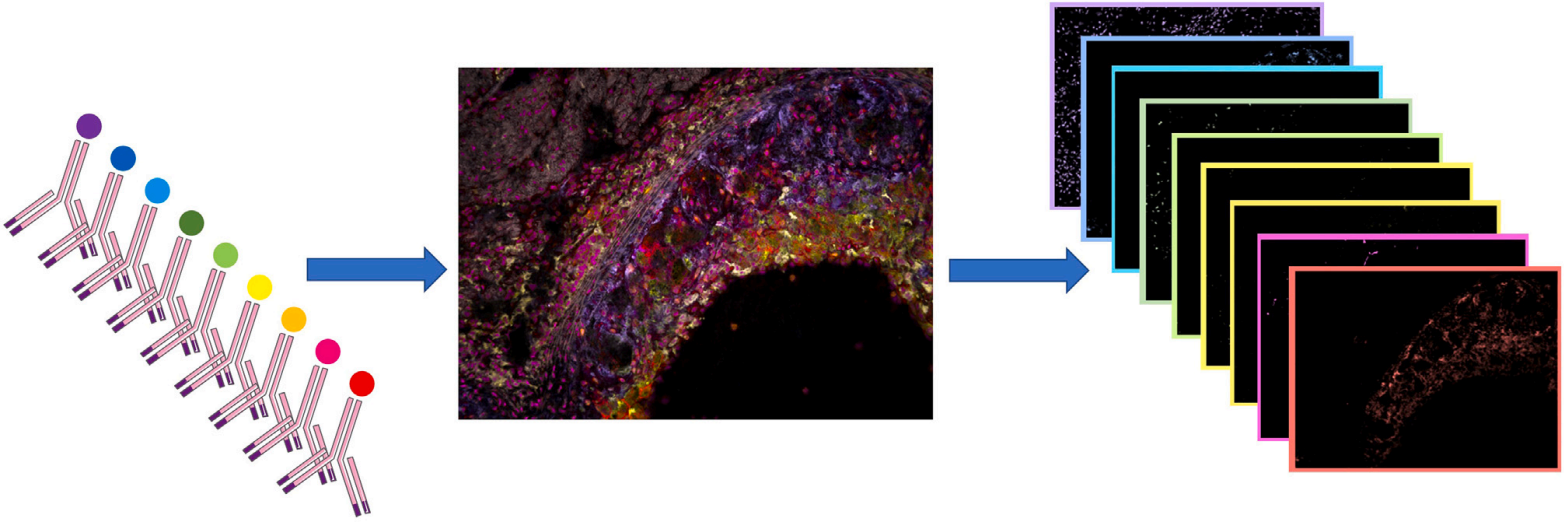
Principle of multispectral imaging and spectral unmixing The tissue section is incubated with a set of dye-coupled antibodies and subsequently imaged using the Nuance FX camera. Spectral unmixing of the composite image will produce an image cube containing the spectral contributions of each dye to the composite image. Using a spectral library, this image cube can be deconvoluted into single grayscale images, each representing one spectral component (dye).

This section describes the preparation steps for multispectral imaging. It details how the fluorescent emission spectrum for each individual fluorochrome must be recorded on tissue sections single-stained with correspondingly labelled antibodies to compose a spectral library. In Figure 1, the principle of multispectral imaging and spectral unmixing can be observed, while Table 1 shows the filter setup used by Goossens et al.*,*^1^ which can still be further adapted to the users’ preferences. When combining a set of fluorochrome-coupled antibodies for multispectral imaging only dyes should be included that are excited by a similar electromagnetic wavelength but whose emissions have different peak intensities and limited spectral overlap.

2. Fix, block and optionally bleach cryosections.
  a. Thaw and air-dry cryosections.
  b. Fix 5 min with dry acetone.
  c. Immerse slides in blocking buffer.
  d. Optional step: Overnight exposure slides in blocking buffer to high-power near-UV light in a cold room (4°C), as we previously described.^1^ An alternative bleaching method is detailed in the troubleshooting section (problem 2).

**Figure 2:**
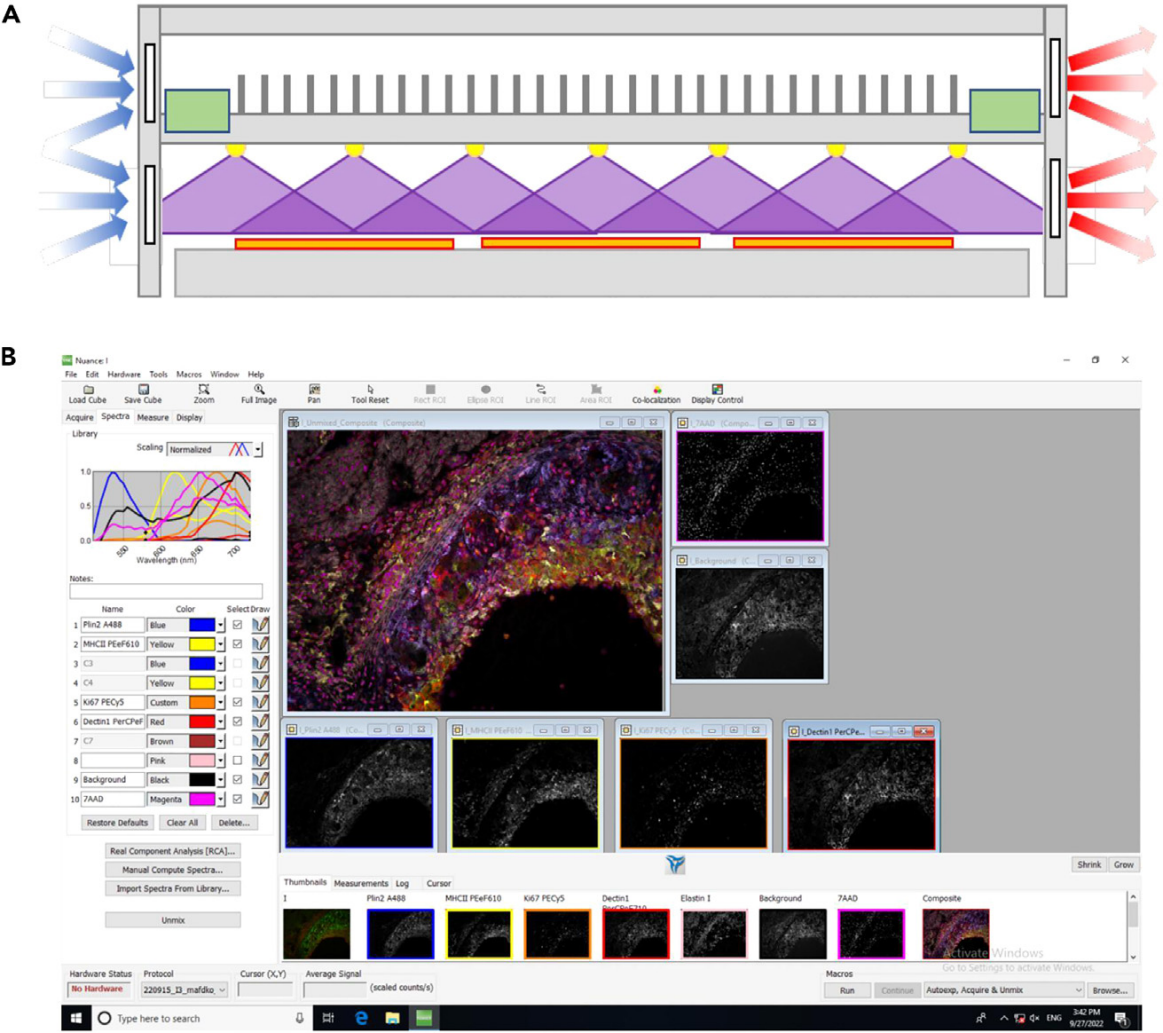
Bleaching of tissue autofluorescence and subsequent immunofluorescent imaging of atherosclerotic plaque tissue (A) Schematic representation of the custom-designed bleaching box, as previously described by us.^1^ This box features a mounting plateau that can accommodate up to 12 tissue slides (orange); near-UV (400 nm) light (purple) by 4 rows of 7 high-power (3 W) LEDs (yellow), evenly distributed over the box; two power sources (green), and both active (fans) and passive (fins) cooling units to prevent the LEDs and tissue sections from overheating. Figure reprinted with permission from Goossens et al., 2022.^1^ (B) Screenshot of the Nuance 3.0.2 software (PerkinElmer), with a spectral library on the left and unmixed images of an image cube representing the spectral library’s individual markers’ fluorescent emission patterns on the right.

**CRITICAL:** In tissues with high autofluorescence, such as liver, brain, and vasculature, we recommend an autofluorescence bleaching step prior to staining. This could for instance be done via the custom-designed bleaching box, depicted in Figure 2A which proved very effective to reduce tissue autofluorescence, increasing the stainings’ signal-to-noise ratio and thereby facilitating spectral unmixing.^1^ This measure makes multispectral imaging also suitable for analysis of previously challenging tissues where high levels of autofluorescence competed with the dyes’ signal.

3. Fluorescent staining.
  a. Dilute antibodies individually in cold blocking buffer containing 7-AAD nuclear dye just prior to staining.
  b. Apply antibody solution on tissue sections and incubate for 4 h in the dark at 4°C.
    ***Note:*** Optionally, tissues can be lined with a PAP pen (Thermo Fisher Scientific) to avoid spill-over of the antibody mix on neighboring tissue sections.
  c. Wash three times with PBS.
  d. Mount coverslip with 50 μL water-based antifade mounting medium (e.g., Prolong Gold, Thermo Fisher Scientific).
  e. Leave the mounted slides to dry for 60 min at room temperature (around 20°C) in the dark before imaging or storing at 4°C.
4. Spectral library composition.
  a. For each stained tissue section, acquire image cubes for each of the excitation filters (Table 1).
    ***Note:*** This is done to visualize the antibody-specific signal and possible bleed-through in different image cubes.
    ***Note:*** Repeat for different antibody concentrations to titrate the optimal dilution.
  b. Define the mean emission spectrum over several pixels with sufficiently high signal-to noise ratio. Check if the detected spectrum corresponds with the theoretical spectrum (as provided by the manufacturer).
    **CRITICAL:** Assess the risk of bleed-through in other spectra and image cubes. For example, when imaging a signal from a dye that is excited at 420 nm and detected at around 650 nm, check whether a similar signal is also detectable upon excitation with 488 nm (problem 3).
  c. Save the spectrum in the spectral library as a reference for this dye.
    ***Note:*** These individually recorded fluorescent emission patterns will be combined in a spectral library, which aggregates the dyes’ spectra for a fixed excitation wavelength with the tissue’s endogenous autofluorescence spectra, recorded on an unstained section.
  d. Unmix the image cube into background and dye-specific signal components to assess the specificity of the newly defined spectrum.
  e. Repeat for the individual dyes and combine the reference spectra into spectral libraries.
    ***Note:*** When a tissue section is stained with a combination of dye-coupled antibodies and imaged using the exact same settings, this spectral library can be used as a reference to computationally deconvolute each of the pixels into their individual fluorescent components, thereby creating a series of images that represent each fluorochrome’s contribution to the overall-measured fluorescence (Figure 1).
    ***Note:*** Contrary to conventional immunofluorescence, where the signal from each fluorochrome is measured as the average intensity in a broad, dye-specific wavelength range, multispectral fluorescence imaging is based on full emission spectra detected for each pixel. In brief, while keeping the excitation constant, multiple images of one field-of-view (FOV) are recorded at narrow, stepwise incrementing wavelength ranges covering the whole visible spectrum. All these two-dimensional images are assembled into a so-called image cube, thereby creating for each individual pixel a third “wavelength” dimension.
    ***Note:*** In order to increase the number of compatible antibodies per staining, spectral scans with different excitation wavelengths can be combined. As an example, Table 1 shows the setup as used in the article by Goossens et al*.*^1^ Different sets of dye-coupled antibodies were excited using four consecutive filter cubes: blue (I3 filter), green (N2.1 filter), UV (A filter) and near-UV light (S Blue Aqua filter, all Leica). Every excitation filter comes with its own spectral library, covering the corresponding excited fluorochromes and tissue autofluorescence.
    ***Note:*** Compare the fluorescent emission pattern of each dye with the theoretical pattern on the manufacturer’s website. Both experimental and manufacturer’s pattern should resemble each other in peak emission, range and shape.
    ***Note:*** The antibody titration and composition of spectral library must be repeated for every tissue type because of tissue-specific autofluorescence that can contribute to the detected fluorochrome-specific spectrum. Thus, separate spectral libraries should be created for every tissue type/species. When designing variations of a library to allow the combination of several custom markers with a fixed set of antibodies, consider assigning the varying slot to a commercially common fluorochrome, such as Alexa Fluor 488.
    **CRITICAL:** Fluorescent dyes strongly differ in emission intensity and the intensities of fluorochromes cannot be scaled individually. Therefore, it is important to stain the markers that show the lowest expression with antibodies coupled to the brightest fluorochromes. In addition, antibody concentrations should be titrated for each of the markers in the panel until similar signal intensities are detected for each, which reduces spectral overlap-based erroneous identification of combined dyes upon unmixing.
    **CRITICAL:** For staining we strongly recommend the use of primary dye-conjugated antibodies to minimize the risk of cross-reaction between antibodies resulting from a limited number of commercially available species during multiplexing. The associated disadvantage is that signal amplification is not possible and that relatively high antibody concentrations are required for detectable signal.
    **CRITICAL:** After the short fixation step, tissue sections must stay hydrated during the whole procedure. Drying will decrease staining quality and increase tissue autofluorescence.
    **CRITICAL:** It is important to keep all variables (e.g., magnification, lamp intensity, exposure time) constant during spectral library composition and multiplex imaging.

**Table 1:** Set of excitation filters and the corresponding emission detection range, exposure time and wavelength incrementation steps employed for multispectral imaging using Nuance 3.0.2 (PerkinElmer) software with a Nuance FX camera (PerkinElmer) mounted on an upright fluorescent microscope (Leica DM4000) equipped with an external light source (Leica EL6000)

Filter cube	Excitation			Emission recorded		
	Band pass (nm)	Dichroic mirror (nm)	Long pass (nm)	Range (nm)	Steps (nm)	Time/step (ms)
I3	450–490	510	515	510–720	5	1100
N2.1	515–560	580	590	580–720	5	700
A	340–380	400	425	420–720	5	3500
S Blue/Aqua	395–415	425	430	420–720	10	1100

### Multispectral imaging

**Timing: 3 days**

After definition of the spectral library and titration of all markers according to step 3 and 4, tissue sections will be stained with the complete antibody panel in one single shot.

***Note:*** This single-shot approach is less time-consuming and laborious compared to sequential staining methods that often compromise antigen and fluorochrome stability as well as tissue integrity over the course of several staining cycles.

***Note:*** The images shown in this protocol were obtained from multiplex staining on murine atherosclerotic plaques.

5. Fix and bleach cryosections.
  a. Repeat procedure as in Step 2.
6. Multiplex antibody staining.
  a. Dilute antibodies included in the complete antibody panel, using the appropriate titration in cold blocking buffer.
    ***Note:*** The complete 15-plex antibody panel and the respective dilutions used for staining of murine atherosclerotic plaque are listed in Table 2.

**Table 2:** Example of a 15-color antibody panel (plus autofluorescence) and their respective dilutions

Antigen-dye	Fluorochrome	Manufacturer	Clone	Dilution
7-AAD	–	Thermo Fisher Scientific	–	1:150
CD11b	Brilliant Blue 700	BD Biosciences	M1/70	1:40
CD44	PE/Cy5	Thermo Fisher Scientific	IM7	1:500
CD47	Brilliant Violet 480	BD Biosciences	Miap301	1:20
CD68	Brilliant Violet 605	BioLegend	FA-11	1:100
CD107b (Mac3)	PerCP/Cy5.5	BD Biosciences	M3/84	1:30
CD206 (MMR)	Brilliant Violet 650	BioLegend	C068C2	1:30
CD205 (DEC-205)	Super Bright 436	Thermo Fisher Scientific	205yekta	1:40
Dectin-1 (Clec7a)	PerCP/eF710	Thermo Fisher Scientific	Bg1fpj	1:30
F4/80	Brilliant Violet 510	BioLegend	BM8	1:40
Ki-67	eFluor 450	Thermo Fisher Scientific	SolA15	1:20
MerTk	Super Bright 702	eBioscience	DS5MMER	1:20
MHC-II (I-A/I-E)	PE/eFluor 610	Thermo Fisher Scientific	M5/114.15.2	1:400
Ly6G	Brilliant Violet 570	BioLegend	1A8	1:20
Perilipin2	Alexa Fluor 488	Novus Biologicals	(polyclonal)	1:20
Elastin	Autofluorescence	–	–	–
  b. Apply the antibody mix on PAP pen-lined tissue sections.
  c. Incubate the slides for 4 h in the dark at 4°C.
  d. Wash 3 times by fully immersing the slides in PBS.
    ***Note:*** If required, incubate another 60 min with secondary antibodies, each specific for not more than one of the primary antibodies, and wash again 3 times with PBS.
  e. Mount coverslip with 50 μL water-based antifade mountant (e.g., Prolong Gold).
  f. Leave slides to dry for 60 min at room temperature (at around 20°C) in the dark.
    **Pause point:** The slides can be stored at 4°C in the dark until the next day for imaging.
  g. Acquire image cubes for every region of interest.
  h. Unmix the image cubes based on their corresponding spectral libraries to obtain single channel grayscale images.
  i. Save each grayscale image as a .jpg file.
    ***Note:*** All images from the same FOV should be saved in a shared folder and should be named consistently to allow automated analysis of larger amounts of data.
    ***Note:*** The individual unmixed grayscale images from a 15-plex antibody panel can be observed in Figure 3. A Schematic representation of the dye combination used for Figures 3A–3P and shown in Table 2 is depicted in Figure 4.
    **CRITICAL:** The tissue section must not be moved in between the acquisition with different excitation filters to allow alignment of all grayscale images.
    ***Note:*** The acquisition of multiple, non-overlapping images from one tissue section ensures a better definition of cell subsets as it reduces the possible bias inherent to a manual selection of a field of view.
    ***Note:*** Depending on the study objective, parallel cell type-specific markers or several phenotypic markers to distinguish subsets of a single cell type, if desired complemented with reporters of the cell’s status (e.g., activation, proliferation, ROS), can be utilized to visualize cells and their cellular context.
    ***Note:*** In case of a high similarity between emission spectra, the use of a negative control and fluorescence-minus-one (FMO) stainings is necessary to validate the correct unmixing of the staining.
    ***Note:*** Documentation of the localization of each field of view on the slide will facilitate retracing it on the subsequently H&E-stained tissue section. This can be done by either annotating a schematic drawing of the tissue section or by annotation on a histology image of an adjacent section.
    ***Note:*** To limit undesired photobleaching, we recommend starting exposure with the image cube requiring the shortest exposure time. Starting with the highest excitation wavelength could also prevent spectral bleaching at lower wavelength due to Stokes’ shift.
    ***Note:*** For optimal visualization, signal-to-noise ratios for each image can be increased in ImageJ. Apply the same settings for all images of the same marker over the different fields of view to avoid bias.

**Figure 3:**
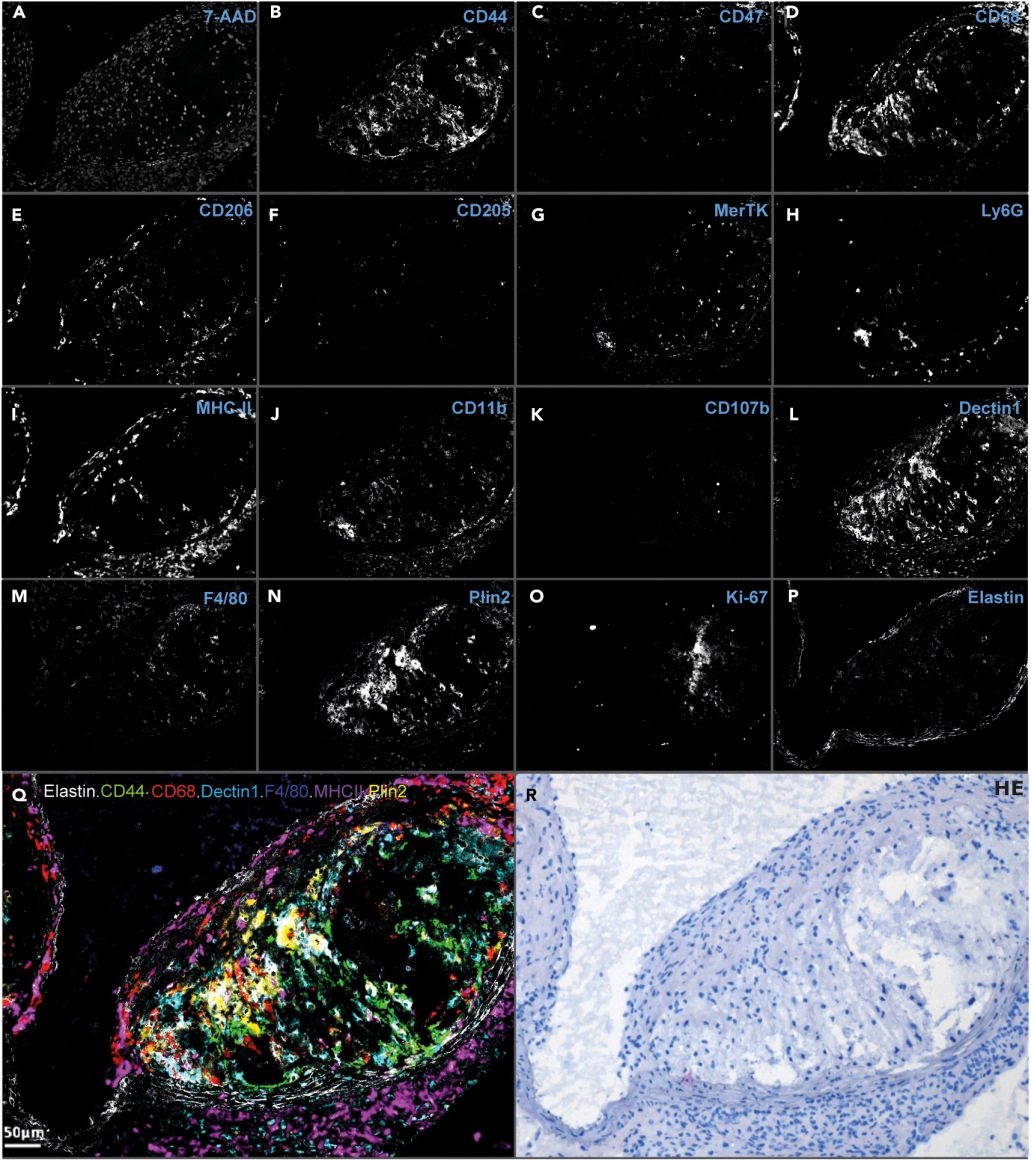
Multispectral imaging of a murine aortic root atherosclerotic plaque section (A–P) Unmixed grayscale images for the individual spectra and thus markers. (Q) A selection of these images was pseudo-colored and merged using ImageJ^4^ to illustrate the relative distributions of these markers over the tissue. Scale bar, 50 µm. (R) H&E image cropped to the same size, orientation, and resolution as the fluorescent images.

**Figure 4:**
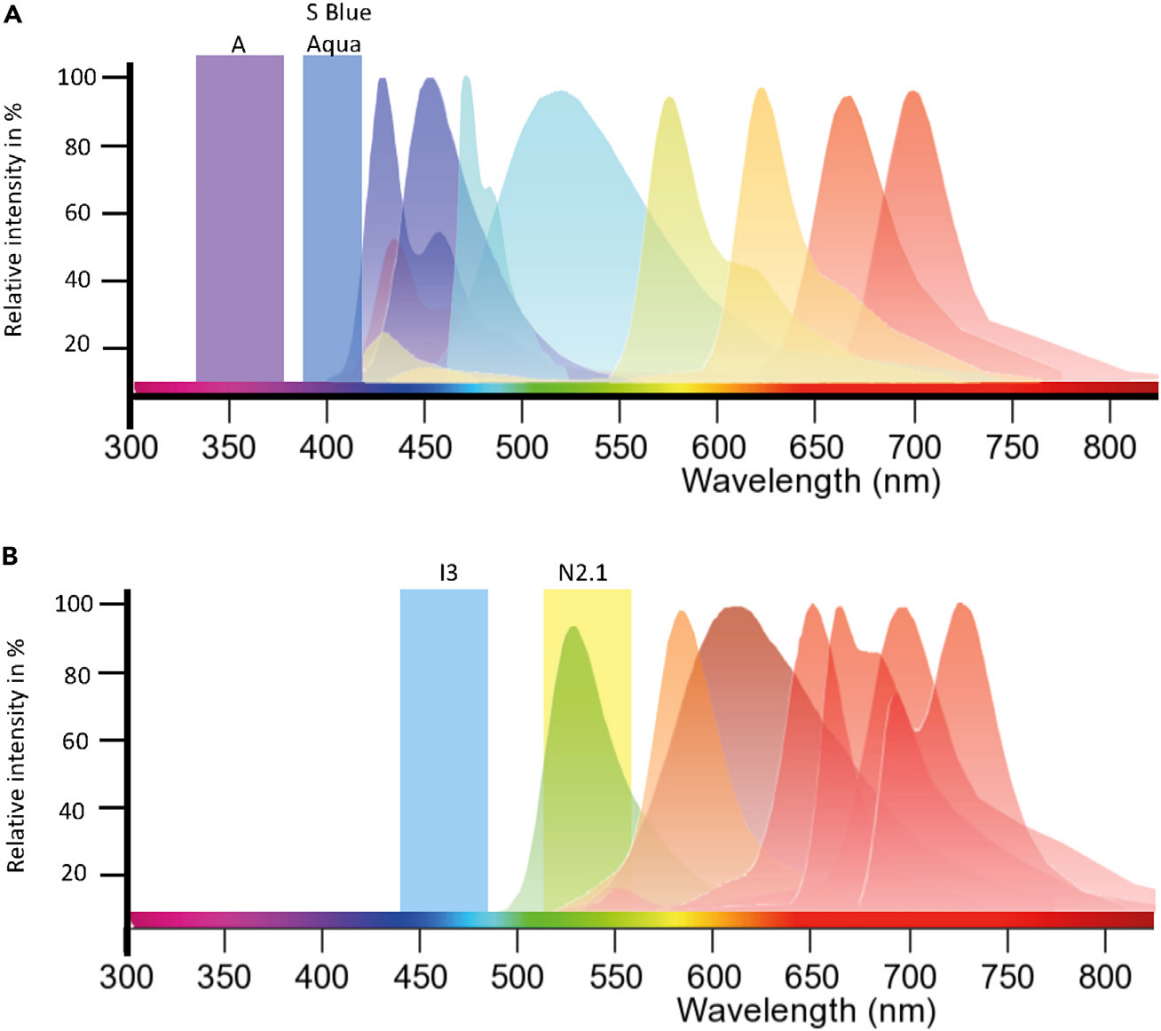
Schematic representation of a dye combination that can be set-up for multispectral imaging (A) A panel of dyes that are excited at 340–380 nm (A) and at 395–415 nm (S Blue/Aqua). The depicted spectra correspond to: Super Bright 436, eFluor 450, BV480, BV510, BV570, BV605, BV650, and SB702 in ascending order. (B) A Panel of dyes that are excited at 450–490 nm (I3) and at 515–560 nm (N2.1). The depicted spectra correspond to: Alexa Fluor 488, PE-eFluor 610, 7-AAD, PerCP-Cy5.5, PE-Cy5, BB700, and PerCP-eFluor 710 in ascending order.
7. H&E staining.
  a. Submerge the fluorescently imaged slides, angled and with the coverslip facing down, in PBS and leave them at 37°C until the cover slip spontaneously detaches (about 20–30 min) to avoid tissue damage during coverslip removal.
  b. Proceed with a standard H&E staining.
  c. Mount the slide with a non-aqueous mounting medium (e.g., Prolong Gold) to allow long-term storage.
  d. Acquire images of the same regions of interest that were fluorescently imaged, but at a lower magnification (e.g., multispectral imaging with a 20× objective, subsequent bright-field imaging with a 10× objective) to later allow cropping it to the same dimensions.

### Multispectral imaging data preparation for downstream analysis

**Timing: 1–5 days**

Here we describe steps that serve as a guideline on how to prepare the acquired multiplex images for downstream analysis. The H&E image acquired in Step 7c will be aligned with the fluorescent images and used for cell segmentation. Afterwards, the mean fluorescent intensity of every dye can be measured for each segmented cell.

8. Alignment of the H&E image and the fluorescent images.
  ***Note:*** Alignment of the H&E image allows the quantification of fluorescence intensities of each marker per individual cell, as well as histopathological characterization of the cells’ morphology, location and microenvironment
  a. Download the ‘AutoRegi_Nuance_HE.mlapp’ app from GitHub.
  b. Install it in MATLAB.
    ***Note:*** Make sure you have previously added the 'Image processing Toolbox’ to your MATLAB.
  c. Run the script, a pop-up window will appear.
  d. Click “load” to open a fluorescent image of the nuclear staining (7-AAD) and the corresponding H&E image file (H&E) from the same tissue section.
  e. Stay in the ‘manually’ panel to align the image manually by selecting landmarks on both fluorescent image and H&E image.
    ***Note:*** Use the ‘auto’ panel for automatic alignment.
    i. Select the ‘selectpoint’, and click ‘run’, a new window will show the fluorescent and bright-field images next to each other.
    ii. Click to place a crosshair on a recognizable landmark in one image and select the corresponding point in the other image.
    iii. Repeat this step several times until minimally 5 landmarks, spread over the field of view, are assigned.
      ***Note:*** To verify accuracy of overlay, click the icon depicting two landmarks to let the script predict additional points. Adjust these manually if necessary.
    iv. Close this pop-up window, the main window will now show a merged image.
    v. Save the cropped H&E image (HE.jpg) if the overlap is accurate.
      ***Note:*** You can save the transformation matrix (tform.mat), or landmarks location by selecting check boxes in the ‘save panel’.
      **CRITICAL:** H&E and fluorescent images must be perfectly aligned (preferred alignment precision: equal or lower than 5 μm) to guarantee that fluorescent signal is cell specific.
      ***Note:*** If you want to perform the alignment of a set of images again, you can load the landmark file from the previous registration or tform.mat into the app by clicking ‘alignwithpoint’ or ‘alingnwithtform’, instead of having to select the landmarks again.
      ***Note:*** You can also align H&E image and 7-AAD image automatically by switching to the ‘auto’ panel. First, adjust the parameters of image processing for both H&E and 7-AAD images. Clicking the ‘processing’ button will display the processed images in the interface. After clicking the ‘registration’, the reregistered image will pop up (Depending on the computer configuration, image size and resolution, this can take some time).
9. Cell segmentation.
  a. Open QuPath.^5^
  b. Open an aligned H&E image (HE.jpg) and set the image type to Brightfield (H&E).
  c. Select the whole image as region of interest or exclude irrelevant areas or artifacts.
  d. Select Analyze > Cell detection > Cell detection.
  e. A settings window will open. For a detailed description of the settings, please refer to the QuPath documentation on this topic.
    i. Toggle off the ‘include cell nucleus’ option and alter the settings until all cells are correctly segmented.
    ii. The cell segmentation can now be copied to ImageJ (Extensions > Image J > Send region to ImageJ).
    iii. In ImageJ, the cell segments will be saved as a mask (Image > Overlay > to ROI manager; select all ROIs in the ROI manager and save them as RoiSet.zip in the corresponding folder) that can be overlaid on each of the fluorescent images to measure the mean dye intensity over each numbered segment (Image > Overlay > from ROI manager).
10. Continue with measuring fluorescent marker (co-)expression per cell segment using standard image analysis programs such as Cell profiler, ImageJ, QuPath or others (see also Goossens et al. *(2022*^1^)).

***Note:*** This fluorescent marker expression per cell can be summarized in one (co-)expression matrix, to be used for downstream heterogeneity analysis and mapping of the cell type or subset distribution.^1^

## Expected outcomes

This protocol helps to set up a robust multiplex imaging pipeline at relatively low costs and with affordable and broadly available laboratory equipment. The above-described multispectral imaging can be used to elucidate the diversity of any cell population in many tissues, disease contexts and species. It has indeed already been used by the authors to map myeloid heterogeneity in a large variety of murine and human healthy and diseased tissues.

The resulting marker expression matrix is suitable for various bioinformatics analyses and can also be combined with (spatial) transcriptomics or spatial Mass Spectrometry-based omics on flanking tissue sections, providing deeper insight into the interplay between cellular heterogeneity and context.

## Limitations

Our protocol relies strongly on antibodies directly labelled with dyes of different Stokes’ shifts that are sometimes not commercially available or not validated for fluorescent microscopy. In addition, the lack of signal amplification implies that lowly-expressed markers may remain undetectable, and the fixed exposure time makes antibody titration a crucial step to obtain comparable fluorescent emission intensities over the different dyes.

Due to the combination of several antibodies in one multiplex staining, application on formalin-fixed paraffin-embedded (FFPE) tissue sections may be challenging due to a diversity in antigen retrieval requirements. Therefore, this protocol is more suitable for staining of cryosections.

The examples shown in this protocol were imaged using a Nuance Fx camera (CRI, later PerkinElmer, next Akoya Biosciences and currently discontinued) and unmixed using the accompanying software (*Nuance software3.0.2, PerkinElmer*). Alternatively, it is possible to use other hyperspectral cameras (e.g., Photon etc.’s Hypercube), tunable filter systems (e.g., Thorlabs’ Kurios) or the lambda scan option readily available on most modern confocal microscopes.

## Troubleshooting

### Problem 1

Some tissues, and especially calcified and/or lipid-rich tissues like bone and atherosclerotic plaque, are challenging to cut with the cryostat. This may lead to inconsistent sectioning and/or cause cutting artifacts, both thwarting the section alignment, or complicate the overlaying of sequential tissue sections.

### Potential solution

Find the optimal section thickness, tissue- and knife temperature for your tissue of interest (e.g., fatty tissues require lower temperatures than collagen-rich tissues). If still challenging, alternative sectioning techniques such as the sticker method, can help to keep tissue sections intact.^6^

### Problem 2

While autofluorescence may hold valuable information by itself (e.g., elastin or lipofuscin) and while its spectrum can be assigned as a separate channel upon unmixing of image cubes, some tissues present important and polychromatic autofluorescence that increases the risk of misassignment of pixels or regions to dye-specific spectra.

### Potential solution

**Figure 5:**
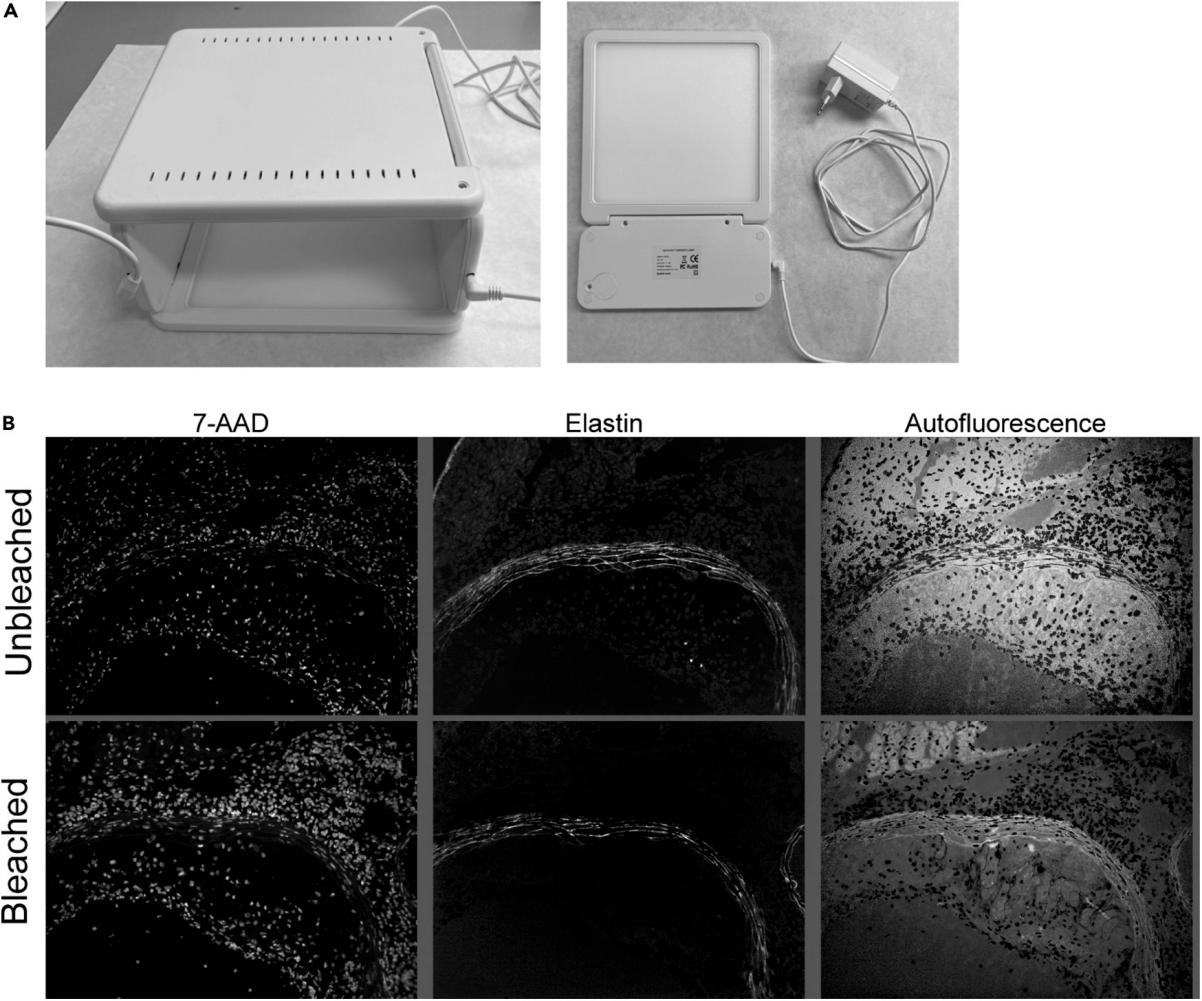
LED panels can be used as alternative to the custom designed bleaching box shown in Goossens et al. (2022) (A) Picture of a single LED panel and two panels assembled as a LED box for bleaching of tissue. (B) Murine atherosclerotic plaques stained with a nuclear dye (7-AAD) and imaged with or without prior bleaching using the LED panels. A 90-min bleaching cycle reduced elastin-specific and general autofluorescence.

We recommend the use of a similar tool for autofluorescence reduction as the custom-build cooled photobleaching device we described before.^1^ Another solution was described by *Du et al*.^7^ This protocol uses LED panels for photobleaching and we demonstrate here that it is an easy-to-set-up alternative to the custom-built UV bleaching device (Figure 5). Briefly, two bright (10.000 LUX each), white light LED panels are positioned above and underneath a translucent container filled with blocking buffer and containing the slides with the tissue sections facing upwards (Figure 5). Sections are bleached for 90 min in a cold room (at 4°C).

Paraffin-embedded tissue sections exhibit stronger autofluorescence, interfering with multispectral imaging. As they resist bleaching solutions better than OCT-embedded sections, the additional use of chemical bleaching protocols based for instance on hydrogen peroxide can be considered.^7^ Alternative ways to reduce autofluorescence include chemical quenching by TrueBlack lipofuscin autofluorescence quencher or Sudan Black B. However, the use of these solutions may also reduce the low intensity signal associated with the use of fluorochrome-coupled antibodies.

### Problem 3

Certain fluorescent dyes have a broad excitation spectrum which may overlap with several filters, resulting in bleed-through.

### Potential solution

When optimizing antibody dilutions in single-stained tissue sections, the imaging protocol must be identical to the one later used to image multiplex stained tissues, meaning that all exposure times, lamp intensity and the order in which the different excitation wavelengths or filters are sequentially applied to the tissue should be maintained. This allows to define such bleed-through of fluorescence as a separate background channel in the affected spectral library. Including too many markers with significant bleed-through may reduce the number of dyes that can be included in the multiplex staining mix.

### Problem 4

Certain fluorescent dyes have almost identical spectral properties, which means that their excitation as well as their emission wavelength have a high resemblance.

### Potential solution

Pre-validation of dye compatibility can be done through using open-access spectral viewer software or websites. Here, dyes can be compared for resemblance of their emission-and excitation spectra. Additionally, some of the spectral viewers (e.g., the Fluorescence SpectraViewer on the Thermo Fisher Scientific website) display a “spill-over” alert that can be used to assess dye compatibility.

A second step of validating the compatibility between two dyes is by performing *fluorescence-minus-one* (FMO) stainings.

**Figure 6:**
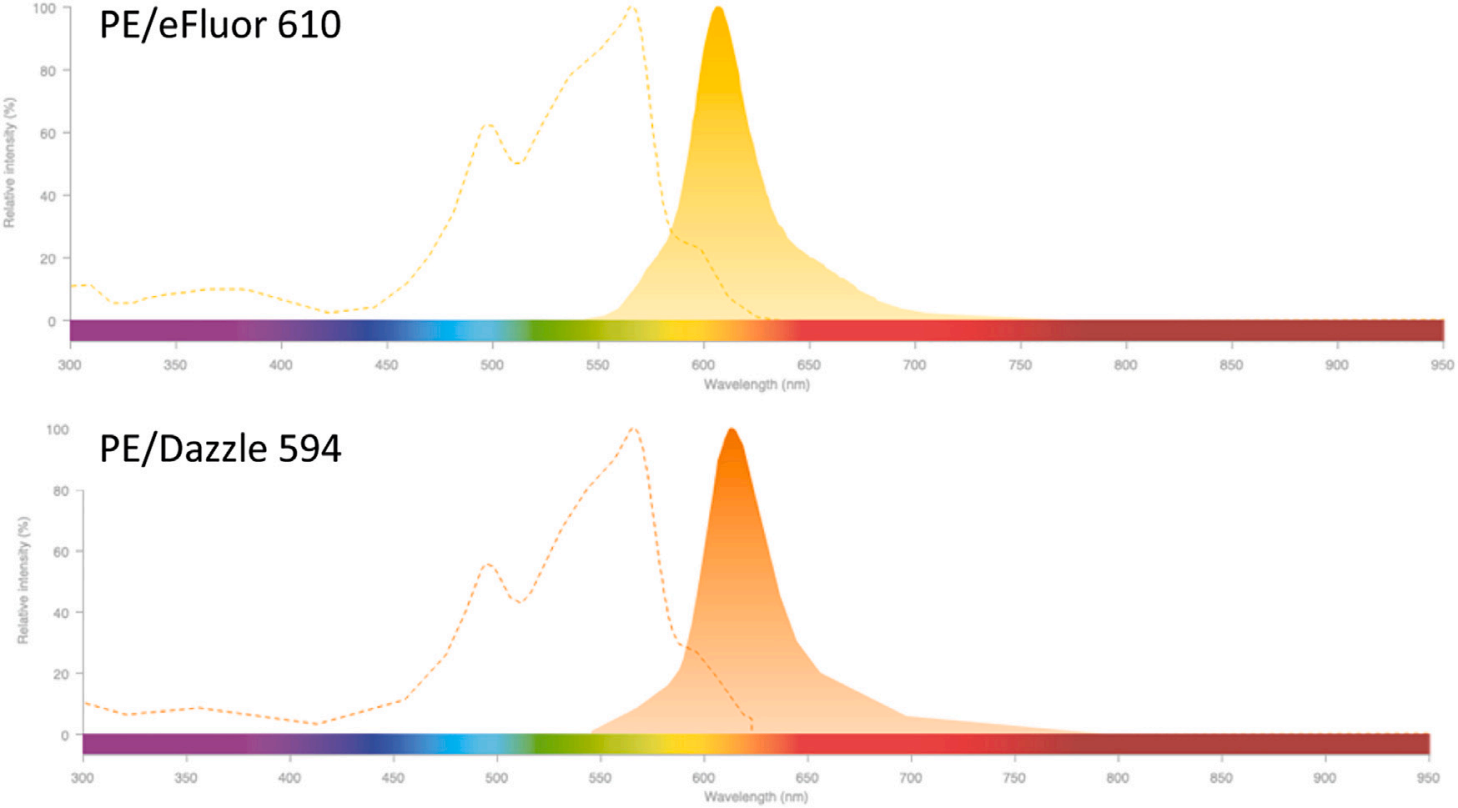
The dyes PE-eFluor 610 and PE/Dazzle 594 are incompatible combinations in a spectral library They show almost identical excitation spectra and have a very similar emission peak and spectra-shape. The figure is adapted from the Fluorescence SpectraViewer (Thermo Fisher Scientific).

An example of incompatible dyes is PE/Dazzle 594 and PE-eFluor 610, who have almost identical excitation as well as emission spectra (Figure 6).

### Problem 5

Cell segmentation is one of the major challenges for multiplex immunofluorescence approaches. Specifically, inaccurate cell segmentation leads to erroneous cell phenotyping and introduces serious bias in the analysis. Problems with accurate cell segmentation can, for example, arise in case of dense cell distribution in tissues (e.g., tumor environments) and high background interference. Thus, cell segmentation remains one of the big challenges in bio-image analysis with currently no failproof solution available.

### Potential solution

A variety of mostly open-access software is available to perform cell segmentation.^8^ Depending on the nature of the tissue that is analyzed, different types of cell segmentation software may be adequate and can be tested to optimize the cell segmentation step. Some of the segmentation software such as StarDist^2^ is also available as a QuPath extension and can thus be easily integrated in the above described workflow.

## Resource availability

### Lead contact

Further information and requests for resources and reagents should be directed to and will be fulfilled by the lead contact, Dr. Pieter Goossens (pieter.goossens@maastrichtuniversity.nl).

### Materials availability

Any request of information on the custom designed bleaching device that was described in this protocol can be directed to Dr. Pieter Goossens (pieter.goossens@maastrichtuniversity.nl).

## Data Availability

The data used in this protocol is available upon request. The code for the alignment of fluorescent and H&E images is available on GitHub (see also key resources table). Any additional information on the analysis shown in Goossens et al.^1^ can be requested from the lead contact, Dr. Pieter Goossens (pieter.goossens@maastrichtuniversity.nl).
